# Complete mitochondrial genome of *Niphades castanea* (Coleoptera: Curculionidae)

**DOI:** 10.1080/23802359.2020.1772683

**Published:** 2020-06-11

**Authors:** Yue Fu, Jiaojun Yu, Xiangliang Fang, Mi Shen, Jun Fu, Yunli Xiao

**Affiliations:** a College of Biology and Agricultural Resources, Huanggang Normal University, Huanggang, China; b Hubei Zhongke Research Institute of Industrial Technology, Huanggang, China

**Keywords:** Curculionidae, *Niphades castanea*, mitochondrial genome, phylogeny

## Abstract

*Niphades castanea* Chao is an important insect pest on many plants which belong to genus *Castanea.* The complete mitochondrial genome of *N. castanea* was sequenced and analyzed. The phylogenetic relationships between *N. castanea* and other 41 species in the family Curculionidae were reconstructed using maximum likelihood (ML) methods based on the concatenated nucleotide sequences, the phylogenetic analysis showed that *N. castanea* is closely related to *Hylobitelus xiaoi*, which is in accordance with the traditional morphological classification.

*Niphades castanea* belong to subfamily Molytinae, its host plants is *Castanea mollissima* and other plants which belong to genus *Castanea*, family Fagaceae (Zhao and Chen 1980; Liu et al. 1995; Zhao et al. 2004; Ji et al. 2008; Xiao et al. 2017).

The specimen in this study was collected from Liuan City (Anhui Province, China) (115°33′24″E, 31°20′24″N) in 2019 and deposited in the Biodiversity Herbarium of Huanggang Normal University (no. HGNU-191201). The species was sequenced using Illumina Miseq platforms and annotated using MITOS web server. PhyloSuite (Zhang et al. 2020) was used for the phylogenetic analyses with several plug-in programs: MAFFT (Katoh and Standley 2013) using ‘–auto’ strategy and codon alignment mode. PartitionFinder2 (Lanfear et al. 2016) was used to select best-fit partitioning schemes and models using AICc criterion. Maximum likelihood phylogenies were inferred using IQ-TREE (Minh et al. 2013; Nguyen et al. 2015). The topology of the trees were visualized and edited in iTOL (Letunic and Bork 2019). The mitogenome sequence of *Carabus changeonleei* (MG253028) was used as the outgroup.

The complete mitogenome of *Niphades castanea* is 17,494 bp in size (GenBank accession number: MT232762). It includes 13 protein-coding genes (PCGs), 22 tRNA genes and 2 rRNA genes, a total of 37 genes, and 1 control region. There are 16 gene overlapping regions appeared, with a total overlapping length of 70 bp, the longest overlapping region (29 bp) located between trnF and nad5. There are 9 intergenic spacers with a total length of 561 bp, ranging from 2 to 452 bp. The longest intergenic was located between trnI and trnQ. The genomic nucleotide composition is A:T:C:G = 39.33%:37.38%:14.02%:9.27%. The total length of 13 PCGs in the mitochondrial genome is 11,133 bp. The initiation codons of PCGs comply with the ATN rule: there are 7 genes (nat2, cox1, cox2, atp8, nad3, nad5, nad6) with ATT as the start codon, 5 genes (atp6, cox3, nad4, nad4l, and cob) with ATG as the start codon, and 1 gene (nad1) with ATA as the start codon. Except for atp8, nad5, and nad1 with TAG as the stop codon, others use TAA, TA(A), or T (AA) as the stop codon. The length of tRNA genes ranked from 63 bp to 71 bp, 1458 bp in total length. The length of 12S rRNA and 16S rRNA are 775 bp and 1272 bp in length, respectively. The control region is 2364 bp, between rrnS and trnI, the AT content is 81.35%.

ML (Figure 1) analyses showed that the subfamily Cryptorhynchinae and Molytinae were clustered together as sister to each other with at 90% bootstrap value of support, Platypodinae and Dryophthorinae were clustered together as sister to each other with at low bootstrap value of support, which is consistent with previous phylogenetic analyses (Marvaldi 1997; Haran et al. 2013; Gillett et al. 2014). The phylogenetic analysis showed that *N. castanea* is closely related to *Hylobitelus xiaoi*, both belong to Molytinae, which is in accordance with the traditional morphological classification.

**Figure 1.**
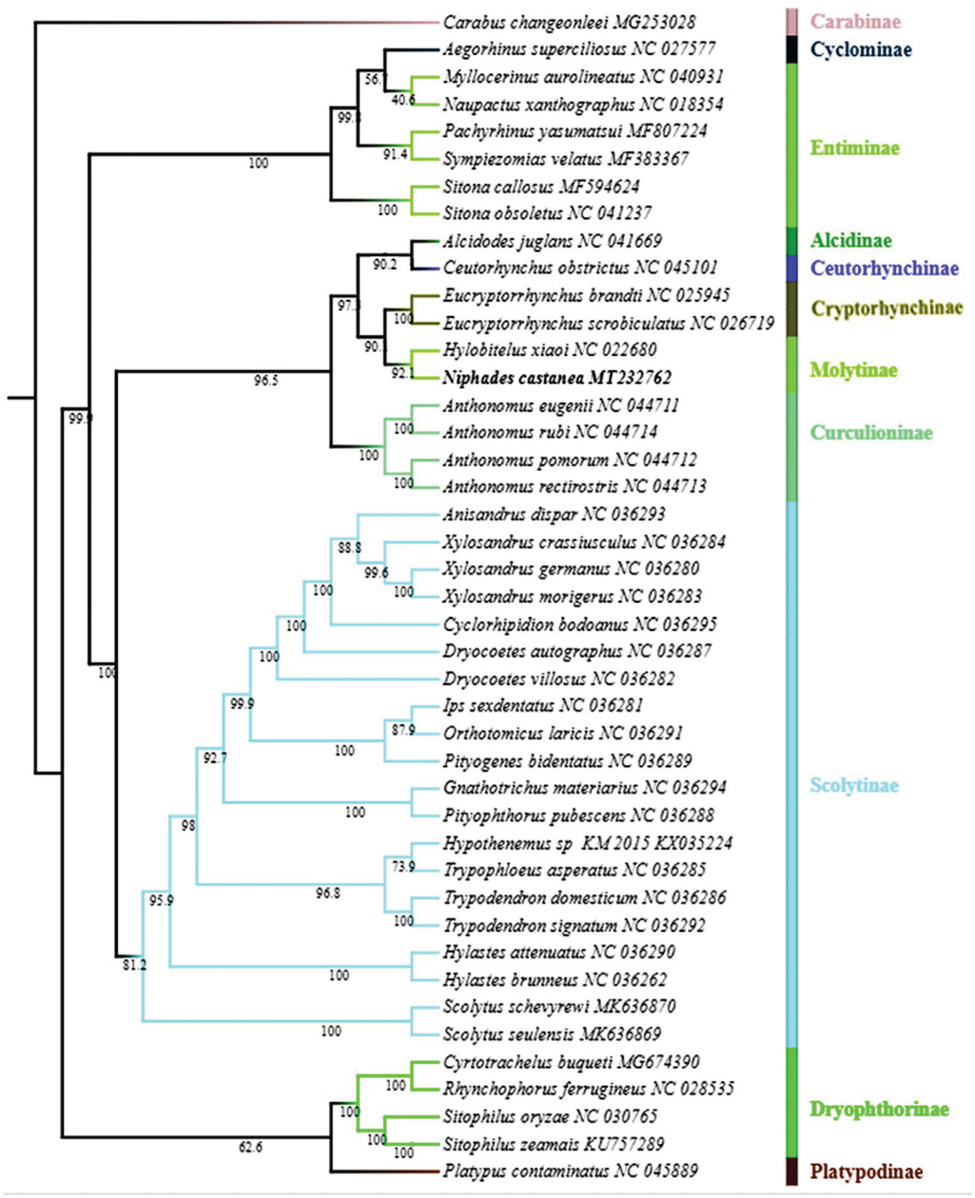
Phylogenetic tree based on 37 genes of mitogenomes of 42 Curculionidae species inferred by maximum likelihood method (ML tree).

## Data Availability

The data that newly obtained at this study are available in the NCBI under accession number of MT232762 (https://www.ncbi.nlm.nih.gov/nuccore/MT232762).

## References

[CIT0001] Gillett CPDT, Crampton-Platt A, Timmermans MJTN, Jordal BH, Emerson BC, Vogler AP. 2014. Bulk de novo mitogenome assembly from pooled total DNA elucidates the phylogeny of weevils (Coleoptera: Curculionoidea). Mol Biol Evol. 31(8):2223–2237. 24803639 10.1093/molbev/msu154 PMC4104315

[CIT0002] Haran J, Timmermans MJ, Vogler AP. 2013. Mitogenome sequences stabilize the phylogenetics of weevils (Curculionoidea) and establish the monophyly of larval ectophagy. Mol Phylogenet Evol. 67(1):156–166. 23319085 10.1016/j.ympev.2012.12.022

[CIT0003] Ji Y, Song XB, Qi RS. 2008. Study on occurrence and control of *Niphades castanes* Chao in Qinba mountainous area. Shaanxi For Sci Technol. (2):91–94.

[CIT0004] Katoh K, Standley DM. 2013. MAFFT multiple sequence alignment software version 7: improvements in performance and usability. Mol Biol Evol. 30(4):772–780. 23329690 10.1093/molbev/mst010 PMC3603318

[CIT0005] Lanfear R, Frandsen PB, Wright AM, Senfeld T, Calcott B. 2016. PartitionFinder 2: new methods for selecting partitioned models of evolution for molecular and morphological phylogenetic analyses. Mol Biol Evol. 34:772–773. 10.1093/molbev/msw260 28013191

[CIT0006] Letunic I, Bork P. 2019. Interactive tree of life (iTOL) v4: recent updates and new developments. Nucleic Acids Res. 47:W256–W259. 10.1093/nar/gkz239 PMC6602468 30931475

[CIT0007] Liu YS, Xu DS, Zhou SY. 1995. Primary report on occurrence regularity and its control of Niphades castanes Chao in Dawu County. Hubei Forest Sci Technol. 29(3):32.

[CIT0008] Marvaldi AE. 1997. Higher level phylogeny of Curculionidae (Coleoptera: Curculionoidea) based mainly on larval characters, with special reference to broad-nosed weevils. Cladistics. 13(4):285–312. 10.1111/j.1096-0031.1997.tb00321.x 34911227

[CIT0009] Minh BQ, Nguyen MA, von Haeseler A. 2013. Ultrafast approximation for phylogenetic bootstrap. Mol Biol Evol. 30(5):1188–1195. 23418397 10.1093/molbev/mst024 PMC3670741

[CIT0010] Nguyen LT, Schmidt HA, von Haeseler A, Minh BQ. 2015. IQ-TREE: a fast and effective stochastic algorithm for estimating maximum-likelihood phylogenies. Mol Biol Evol. 32(1):268–274. 25371430 10.1093/molbev/msu300 PMC4271533

[CIT0011] Xiao YL, Zhang F, Xu YX, Zhong YL. 2017. Discrimination of two chestnut fruit borer weevils (Coleoptera: Curculionidae). Plant Protect. 43(4):104–109.

[CIT0012] Zhang D, Gao F, Jakovlić I, Zou H, Zhang J, Li WX, Wang GT. 2020. PhyloSuite: An integrated and scalable desktop platform for streamlined molecular sequence data management and evolutionary phylogenetics studies. Mol Ecol Resour. 20(1):348–355. 31599058 10.1111/1755-0998.13096

[CIT0013] Zhao GR, Yang CC, Cai YP. 2004. Investigation on life cycle of snow flake curculio. J Anhui Agric Univ. 31(4):484–487.

[CIT0014] Zhao YC, Chen YQ. 1980. Economic insect fauna of China. Fasc. 20. Coleoptera, Curculionidae(I). Beijing: Science Press.

